# Assessing coping strategies among intimate partner violence victims

**DOI:** 10.1192/j.eurpsy.2024.1682

**Published:** 2024-08-27

**Authors:** F. Tabib, F. Guermazi, S. Hentati, D. Mnif, I. Feki, I. Baati, J. Masmoudi

**Affiliations:** Department of psychiatry A, UHC Hedi Chaker, Sfax, Tunisia

## Abstract

**Introduction:**

Intimate partner violence (IPV) is a major source of perceived stress for the women who suffer from it. To cope, they tend to implement multiple coping strategies depending on a number of contextual factors including, among others, the severity and frequency of abuse, the duration of the relationship, and available resources such as social support and financial resources.

**Objectives:**

To study the coping strategies used by women who are victims of IPV.

To study the factors associated with coping strategies among these women.

**Methods:**

We conducted a descriptive and analytical cross-sectional observational study, carried out over a 10-month period from March 2021 to December 2021, among female victims of IPV consulting psychiatric emergencies at UHC Hedi Chaker, Sfax, Tunisia for medical expertise at the request of the court.

The Brief-COPE is a 28-item self-assessment questionnaire designed to measure coping with a stressful life event. It can be divided into 3 subscales: problem-focused, avoidance-focused, and emotion-focused.

**Results:**

The total number of participants was 120 with an average age of 37.27 years. The majority had secondary education or less (62.5%), were professionally active (53.3%), and were financially dependent on their partners (26.7%). As for the women’s clinical characteristics, 19.2% were under psychiatric care and 15% had attempted suicide (SA). Almost all the women surveyed (99.2%) had reported at least one previous incident of IPV. These incidents were daily in 60.5% of cases. Emotional violence was severe in 75.8% of women.

The emotion-focused strategy was the most widely used, with a mean score (29.68) on the Brief cope scale. It was correlated with the absence of a personal psychiatric history (p=0.02), the absence of SA (p=0.036), and the occasional frequency of IPV (p=0.037). The scores for problem-focused coping and avoidance-focused coping are 19.3 and 17.24 respectively. Avoidance-focused coping was negatively correlated with the presence of severe emotional abuse.

**Conclusions:**

The most used strategy by our population was the emotion-focused strategy, with a relatively high average score compared to the other strategies. Indeed, it may be an extremely effective strategy for recovering from a traumatic event, through actions designed to help these women manage and relieve their psychological distress and reduce its negative impact.

**Disclosure of Interest:**

None Declared

